# Lifetime Socioeconomic Status, Cognitive Decline, and Brain Characteristics

**DOI:** 10.1001/jamanetworkopen.2024.61208

**Published:** 2025-02-21

**Authors:** Kristin R. Krueger, Pankaja Desai, Todd Beck, Lisa L. Barnes, Jerenda Bond, Charles DeCarli, Neelum T. Aggarwal, Denis A. Evans, Kumar B. Rajan

**Affiliations:** 1 Rush Institute for Healthy Aging, Rush University Medical Center, Chicago, Illinois; 2 Rush Alzheimer’s Disease Center, Rush University Medical Center, Chicago, Illinois; 3 Department of Neurology, University of California at Davis, Davis

## Abstract

**IMPORTANCE:**

Socioeconomic status (SES) has an important association with cognitive function and structural brain indices. Identifying the nature of this association will guide strategies for improving health equity.

**OBJECTIVE:**

To test the longitudinal associations of SES with cognitive decline and brain characteristics and to examine whether these associations differ between Black and White individuals.

**DESIGN, SETTING, AND PARTICIPANTS:**

Participants aged 65 years old or older were recruited for this population-based cohort study from 4 communities on the south side of Chicago, Illinois. At-home interviews were conducted between 1993 and 2012. The data were analyzed in April 2024.

**MAIN OUTCOMES AND MEASURES:**

Outcome measures were level and change in global cognition and 4 individual tests. Three magnetic resonance imaging (MRI) measures of the brain included total brain volume, hippocampal volumes, and white matter hyperintensities (WMH). Childhood SES was assessed using parental education levels, father’s occupation, and childhood finance rating. Adulthood SES was assessed using the participants’ education, occupation, and income. Lifetime SES was assessed on the basis of the mother’s education, childhood SES, and participants’ occupation and income.

**RESULTS:**

Of the 7303 participants (mean [SD] age, 72.3 [6.3] years; 4573 female participants [63%]), 4581 (63%) were non-Hispanic Black, and 2722 (37%) were non-Hispanic White. SES was higher for White individuals compared with Black individuals in childhood, adulthood, and across the lifespan. Higher lifetime SES was associated with better global cognitive functioning at baseline (estimate, 0.337; 95% CI, 0.317 to 0.357; *P* < .001) but not with decline over time (estimate, 0.003; 95% CI, −0.001 to 0.006; *P* = .10). Higher lifetime SES was associated with a better baseline score on the Mini-Mental State Examination (estimate, 0.281; 95% CI, 0.261 to 0.302; *P* < .001) and a slower decline for all participants (estimate, 0.012; 95% CI, 0.008 to 0.016; *P* < .001). In a subset of 933 participants who underwent MRI, there was an association between lifetime SES and healthier brain structures, as measured by total brain volume (estimate, 3.18; 95% CI, 0.20 to 6.17; *P* = .04) and WMH burden (estimate, −0.11; 95% CI, −0.21 to −0.01; *P* = .03).

**CONCLUSIONS AND RELEVANCE:**

In this cohort study, SES, mainly in adulthood, was associated with a person’s cognitive status and brain structure, resulting in a discrepancy in cognitive status over time. These findings point to a need for interventions that improve SES throughout the lifespan, particularly for Black individuals, who had lower SES than White individuals.

## Key Points

**Question:**

Is socioecomonic status (SES) across the lifespan associated with cognitive decline and brain characteristics?

**Findings:**

In this population-based cohort study of 7303 participants, SES was lower throughout the lifespan for Black individuals compared with White individuals. Higher SES, mainly in adulthood, was associated with better cognitive function, less cognitive decline, and better brain characteristics, including white matter hyperintensities and total brain volume.

**Meaning:**

These findings suggest that SES is an essential factor in brain health disparities and point to a need for interventions that improve SES throughout the lifespan.

## Teaser

This cohort study examines associations of socioeconomic status across the lifespan with cognitive decline and brain characteristics in Black and White individuals in Chicago, Illinois.

## Introduction

Understanding the association of socioeconomic status (SES) with cognitive functioning is essential for addressing brain health inequities. Although SES is a well-established risk factor for several other health conditions,^1,2,3^ the study of SES as a factor in cognitive aging has recently gained traction.^4^ Thus far, the findings on SES have consistently revealed that lower SES is associated with a lower level of cognitive functioning in older adults,^4,5^ thereby affecting dementia risk. Conversely, the results have been less consistent regarding an association between SES and a decline in cognitive functioning. One study^5^ revealed that higher SES factors in childhood were associated with a slower decline in old age, but midlife SES factors were not. Several other studies^6,7,8,9,10^ failed to find an association between SES and the rate of cognitive decline.

Nevertheless, at least one study^11^ reported that higher late-life adult income was associated with slower cognitive decline. However, another group of researchers indicated that, among individuals with low levels of education, different associations exist between SES and cognitive decline, depending on the cognitive domain.^12^

Identifying the contributions of SES and racial and ethnic background to higher rates of cognitive disorders among non-Hispanic Black individuals continues to be critical.^13,14^ By and large, racial and ethnic disparities have been found to be cross-sectional and not longitudinal. The higher prevalence of dementia among non-Hispanic Black (hereafter, Black) individuals than non-Hispanic White (hereafter, White) individuals, likely because of having lower test performance and crossing the threshold into a disease state at earlier ages, has been a consistent finding in our population-based studies.^6^ That is, although Black individuals have a greater prevalence of dementia than White individuals, the rate of cognitive decline is similar for both groups.^15^ The more complicated associations among racial and ethnic background, SES, and cognitive functioning are still not fully understood.

It has been hypothesized that any association of SES with cognitive functioning may be related to changes in brain structure; however, the literature has failed to provide consistent findings between SES variables and brain structure differences. For example, one study^16^ revealed that education level was not associated with brain structure in those with mild cognitive impairment, but it was positively associated with enhanced brain structure in healthy controls and was negatively associated with brain structure in patients with Alzheimer disease. Another study^17^ reported that the influence of SES varied by race and ethnicity. Specifically, Black individuals with low SES had greater white matter (WM) pathology, whereas White individuals with high SES had greater total brain volume.^17^ Another study revealed that lower-status occupations in midlife and lower SES positions were associated with faster hippocampal atrophy.^18^ A few studies have examined SES at the neighborhood level, finding that disadvantaged neighborhood variables are associated with reduced brain structure^19^ and Alzheimer disease neuropathology.^20^ Finally, a recent review of the literature on SES, brain volume, and cognitive ability from the US and Europe concluded that the current literature, “…underscore[s] that SES has no uniform association with, or impact on, the brain and cognition.”^21^

There are numerous challenges in examining the association of SES with cognitive aging, which then affects research findings. SES has been defined differently across studies and measured at different times (eg, childhood, midlife, and late life). Occupational prestige, income, and wealth are distinct yet related concepts that are not fully defined or applied consistently. SES can be addressed at the neighborhood level,^5,19^ an individual or household level,^9^ or both.^7^ Despite the various ways to measure SES, it is an essential factor for brain health and likely represents a pattern of resources.^4^ Our study examined the association of SES, as defined by a combination of income and education, with cognitive function and cognitive decline in a diverse group of older adults. We looked at the outcomes of household SES among older adults stratified by race in childhood, adulthood, and over the lifespan. Finally, we examined the associations between SES and brain characteristics, as measured by magnetic resonance imaging (MRI).

## Methods

### Participants

All the participants were surveyed as part of the Chicago Health and Aging Project, a population-based cohort study. The participants were residents of 1 of 4 community areas on the south side of Chicago, Illinois: Beverly, Mount Greenwood, Washington Heights, or Morgan Park. The participants were required to be at least 65 years old to enter the study. Data were collected from the participants’ homes every 3 years from 1993 to 2012. Race was self-identified according to categories used in the 1990 US Census. The number of individuals who were a race other than Black or White was very low, so we refer only to these 2 categories. The institutional review board of the Rush University Medical Center approved all study measures and protocols, and all participants provided written informed consent for all study aspects. We have carefully consulted the Strengthening the Reporting of Observational Studies in Epidemiology (STROBE) reporting guidelines and checklist in editing this report.

### Cognitive Functioning

Cognitive function was measured using a battery of 4 tests that were validated in multiple studies over more than 20 years. The battery consists of the following tests: (1) immediate recall measured by the East Boston Test, (2) delayed recall measured by the East Boston Test,^22,23^ (3) perceptual speed measured by the Adapted Digit Symbol Test,^24^ and general cognitive function measured by the Mini-Mental State Examination (MMSE).^25^ We standardized each test score to a *z* score by using the baseline mean and SD of the Chicago Health and Aging Project population. As such, the mean of our converted scores equals 0, and the SD is 1. We then averaged the 4 tests into a global composite score to reduce ceiling and floor effects, allowing us to determine our sample’s range of abilities. Additionally, this procedure allows us to examine cognitive function over time, hence fulfilling the objective of our longitudinal study.^26^ We combined both the immediate and delayed recall scores of the East Boston Test to form a memory score and used the Adapted Digit Symbol Test as a speed score. This battery was administered once per 3-year cycle.

### SES Measurement

SES in childhood was measured using 4 components based on self-reports: (1) father’s education, (2) mother’s education, (3) father’s occupation, and (4) childhood finances rating. SES in adulthood was measured using 3 components related to the participant: education, occupation, and income. Lifetime SES was then formed to reflect SES components in childhood and adulthood and was composed of (1) mothers’ education, (2) occupation (determined with the Hauser and Warren Socioeconomic Index, a 4-digit-number,^27^ which is based on occupation performed for most of the work history and educational attainment), (3) childhood SES, and (4) income. To determine income, a participant was presented with a list of items that provided a range of possible income levels to indicate which one best matched their current income.

Educational levels were determined according to the number of academic years completed and recorded continuously (eg, 1-30 years). To obtain a childhood finance rating, the participants chose 1 of the following categories to describe their family’s financial situation when they were a very young child: 1, very poor; 2, somewhat poor; 3, approximately average; 4, somewhat well off; or 5, very well off. For each SES determination (childhood and adult), the individual component scores were converted into *z* scores and then averaged to create a continuous variable.

### MRI Evaluations

The MRI sequences of total brain volume, hippocampal volume, and WM hyperintensity (WMH) were obtained through fluid-attenuated inversion recovery, spoiled gradient-recalled sequence with an echo time minimum, and double-spin^28^ via a 1.5-T scanner (Genesis Signa and Signa Excite; both from GE Medical Systems). A hypothetical line connects the anterior and posterior commissures so the scans can be oriented parallel to them. They were then transferred digitally to the IDeA laboratory, where they were processed and analyzed.

Skull removal was completed via an atlas-based method and quality control by laboratory staff.^29^ The brain images were then nonlinearly registered by a cubic B-spline deformation^30^ to a synthetic brain image adapted for brain images of individuals older than 60 years. We corrected for field inhomogeneity bias with a template-based iterative method.^31^ The most consistent outputs using the native-space images along with a model of image smoothness^32^ were produced with an expectation-maximization algorithm that iteratively refines the segmentation estimates. Markov random field inference was computed via an adaptive priors model and refined until convergence. Regions of interest were based on expert definitions found in the literature, including Das et al,^33^ the Desikan-Killiany Atlas from Freesurfer,^34^ and Brodmann areas. Cortical thickness was estimated via the registration-based method of Das et al,^33^ which consists of initial probabilistic segmentation of gray matter (GM), WM, and cerebrospinal fluid after intensity inhomogeneity correction^32^ and segmentation.^35^ A greedy diffeomorphic registration was used to match the GM and WM segments or reach a maximum displacement of 6 mm. For each voxel on the GM-WM boundary, the thickness is computed as the distance moved under the registration transformation and propagated across the GM mask.

### Statistical Analysis

We performed analyses to describe participant attributes in our study at baseline. We conducted linear mixed-effects models to examine the longitudinal association between global cognitive function and SES in childhood, adulthood, and lifetime, as stratified by race. These models provide tests for the associations of SES with the baseline level of cognition and the annual rate of change in cognition. The models were adjusted for age and sex and included random slopes and intercepts. We repeated these analyses for episodic memory, speed score, and MMSE cognitive tests. Statistical significance was defined as *P* < .05, calculated with Welch 2-sample *t* test or Pearson χ^2^ test.

In a subset of participants who underwent MRI, we explored the cross-sectional associations between childhood SES, adulthood SES, lifetime SES, and 3 different brain regions via linear regression models. The 3 brain indices were (1) total brain volume, (2) hippocampal volume, and (3) WMH. A log transform was used because of the highly skewed distribution of WMH. The residual correction method was used to correct for variation because of individual differences in cranial volume. Each MRI variable was regressed on cranial volume, and the resulting residuals were then analyzed, allowing for an effect for the MRI measurement independent of total cranial volume. All statistical analyses were performed in SAS statistical software version 9.4 (SAS Institute) in April 2024.^36^

## Results

**Figure 1.**
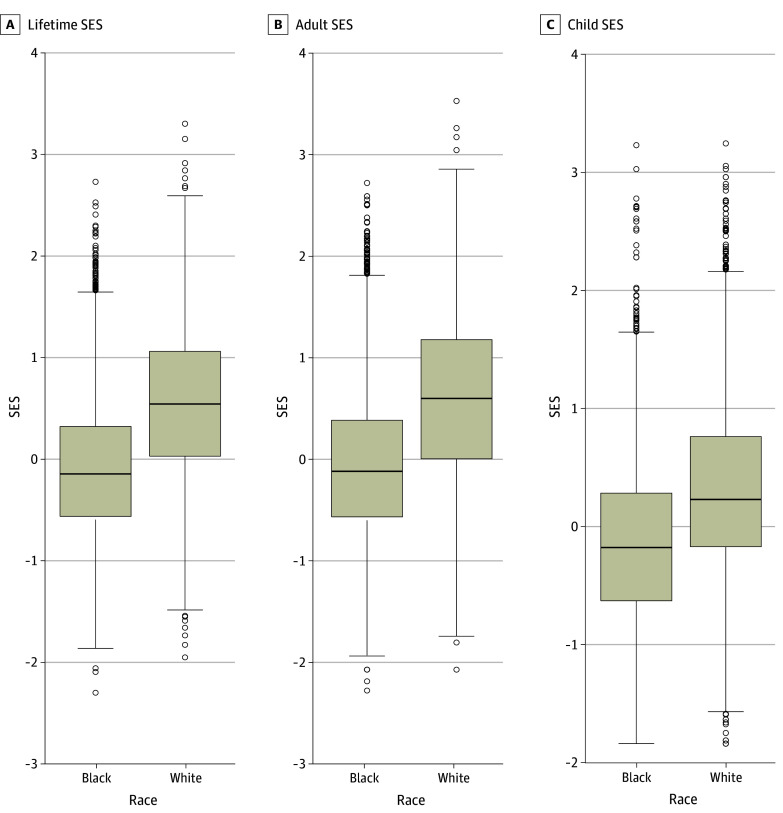
Lifetime, Adult, and Childhood Socioeconomic Status (SES) by Race Box plots depict SES scores. Lines within boxes denote medians, tops of boxes denote 75th percentiles, bottoms of boxes denote 25th percentiles, and circles denote outliers. Whiskers denote 75th percentile + 1.5 × IQR and 25th percentile – 1.5 × IQR.

Of the 7303 participants (mean [SD] age, 72.3 [6.3] years; 4573 female participants [63%]), 4581 (63%) were Black, and 2722 (37%) were White. They had a mean (SD) of 12.5 (3.6) years of education. The mean (SD) global cognition score was 0.30 (0.72) based on a standardized *z* score, as described previously.^26^ Table 1 lists the participant characteristics overall and by race, demonstrating significant differences between the 2 groups for most characteristics. As shown in the box plots in Figure 1, SES in childhood, adulthood, and lifetime was statistically significantly greater for White individuals than Black individuals.

**Table 1.** Characteristics of Study Cohort

Variable	Mean (SD)			*P* value^a^
	Overall (N = 7303)	Non-Hispanic White (n = 2722)	Non-Hispanic Black (n = 4581)	
Age, y	72.3 (6.3)	74.1 (6.9)	71.2 (5.6)	<.001
Sex, No. (%) of participants				
Female	4573 (63)	1674 (61)	2899 (63)	.13
Male	2730 (37)	1048 (39)	1682 (37)	
Education, y	12.5 (3.6)	14.0 (3.2)	11.6 (3.4)	<.001
Socioeconomic status score				
Childhood	0.04 (0.73)	0.32 (0.74)	−0.13 (0.66)	<.001
Adult	0.23 (0.83)	0.66 (0.79)	−0.03 (0.75)	<.001
Lifetime	0.19 (0.77)	0.61 (0.72)	−0.06 (0.67)	<.001
Test scores				
Global cognition	0.30 (0.72)	0.56 (0.62)	0.14 (0.73)	<.001
Episodic memory	0.29 (0.83)	0.49 (0.75)	0.18 (0.86)	<.001
Perceptual speed	0.37 (0.94)	0.84 (0.81)	0.08 (0.90)	<.001
Mini-Mental State Examination	0.28 (0.67)	0.47 (0.53)	0.17 (0.72)	<.001
Time in study, y	7.8 (4.3)	7.5 (4.3)	8.0 (4.3)	<.001

^a^ Calculated with Welch 2-sample *t* test or Pearson χ^2^ test.

### SES in Adulthood

**Figure 2.**
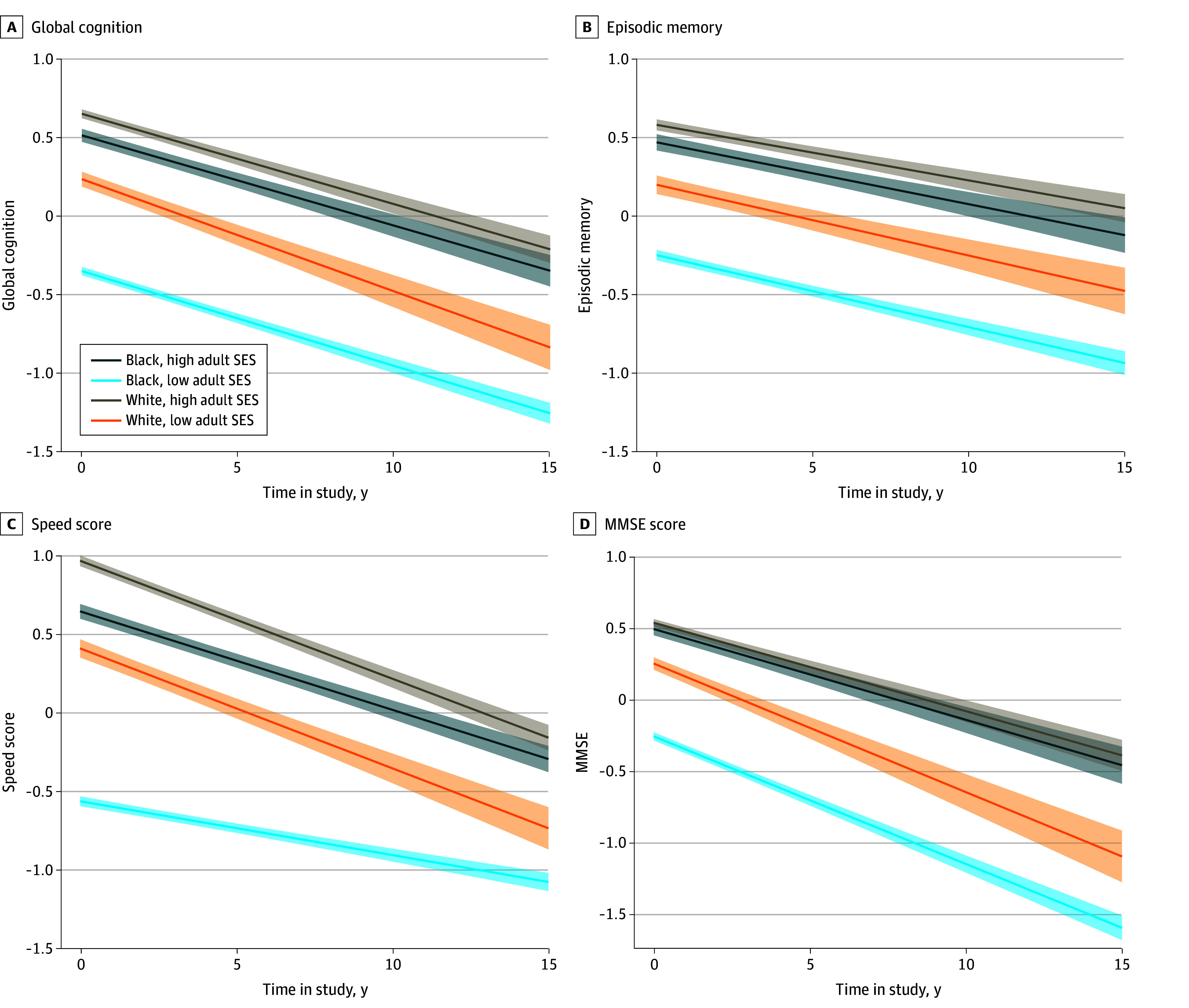
Longitudinal Changes Among Non-Hispanic Black and Non-Hispanic White Individuals by Adult Socioeconomic Status (SES) (10th and 90th percentiles) Lines denote means, and shaded areas denote 95% CIs. MMSE indicates Mini-State Mental Examination.

As indicated in Table 2, higher adult SES was associated with better global cognitive functioning for all participants at baseline (estimate, 0.311; 95% CI, 0.292 to 0.330; *P* < .001) and with changes over time in White individuals (estimate, 0.008; 95% CI, 0.002 to 0.013; *P* = .01) but not Black individuals (estimate, 0.001; 95% CI, −0.003 to 0.005; *P* = .76). Specifically, for each 1-unit increase in adult SES, the annual decline in global cognition slowed by 0.008 (95% CI, 0.002-0.013) for White individuals, or 10% slower than the average. Figure 2A illustrates this difference in global cognition between high (90th percentile) and low SES (10th percentile) in adulthood for both groups.

**Table 2.** SES and Cognitive Function at Baseline and Over Time^a^

Variable	All participants		Non-Hispanic African-American individuals		Non-Hispanic White individuals	
	Estimate (95% CI)^b^	*P* value	Estimate (95% CI)^c^	*P* value	Estimate (95% CI)^c^	*P* value
Global cognition						
No. of participants	7303		4581		2722	
Child SES	0.019 (−0.001 to 0.04)	.06	0.033 (0.006 to 0.061)	.02	0.013 (−0.016 to 0.042)	.37
Child SES × time	−0.001 (−0.004 to 0.002)	.57	−0.001 (−0.005 to 0.004)	.81	−0.003 (−0.009 to 0.003)	.28
Adult SES	0.311 (0.292 to 0.330)	<.001	0.385 (0.360 to 0.410)	<.001	0.187 (0.158 to 0.215)	<.001
Adult SES × time	0.003 (0.000 to 0.006)	.06	0.001 (−0.003 to 0.005)	.76	0.008 (0.002 to 0.013)	.01
Lifetime SES	0.337 (0.317 to 0.357)	<.001	0.424 (0.398 to 0.450)	<.001	0.200 (0.171 to 0.230)	<.001
Lifetime SES × time	0.003 (−0.001 to 0.006)	.10	0.001 (−0.003 to 0.005)	.66	0.005 (0.000 to 0.011)	.07
Episodic memory						
No. of participants	7266		4558		2708	
Child SES	−0.003 (−0.028 to 0.022)	.82	0.005 (−0.029 to 0.040)	.77	−0.003 (−0.039 to 0.032)	.87
Child SES × time	0.001 (−0.003 to 0.005)	.54	0.000 (−0.005 to 0.005)	.94	0.002 (−0.005 to 0.008)	.62
Adult SES	0.270 (0.247 to 0.294)	<.001	0.327 (0.295 to 0.358)	<.001	0.175 (0.139 to 0.211)	<.001
Adult SES × time	0.003 (−0.001 to 0.007)	.10	0.002 (−0.003 to 0.007)	.38	0.004 (−0.002 to 0.010)	.19
Lifetime SES	0.281 (0.256 to 0.305)	<.001	0.346 (0.313 to 0.378)	<.001	0.178 (0.141 to 0.215)	<.001
Lifetime SES × time	0.004 (0.000 to 0.008)	.04	0.003 (−0.002 to 0.008)	.27	0.005 (−0.001 to 0.011)	.13
Speed score						
No. of participants	7267		4355		2604	
Child SES	0.057 (0.033 to 0.081)	<.001	0.084 (0.053 to 0.115)	<.001	0.32 (−0.003 to 0.068)	.75
Child SES × time	−0.002 (−0.006 to 0.001)	.14	−0.001 (−0.004 to 0.003)	.80	−0.007 (−0.012 to −0.001)	.18
Adult SES	0.423 (0.401 to 0.446)	<.001	0.531 (0.502 to 0.560)	<.001	0.248 (0.212 to 0.283)	<.001
Adult SES × time	−0.008 (−0.011 to −0.005)	<.001	−0.013 (−0.017 to −0.010)	<.001	0.003 (−0.003 to 0.008)	.38
Lifetime SES	0.476 (0.452 to 0.500)	<.001	0.607 (0.577 to 0.636)	<.001	0.270 (0.233 to 0.306)	<.001
Lifetime SES × time	−0.009 (−0.013 to −006)	<.001	−0.014 (−0.017 to −0.010)	<.001	−0.002 (−0.008 to 0.004)	.49
Mini-Mental State Examination						
No. of participants	7612		4886		2726	
Child SES	0.019 (−0.002 to 0.040)	.07	0.032 (0.003 to 0.061)	.03	0.019 (−0.007 to 0.045)	.16
Child SES × time	−0.002 (−0.006 to 0.003)	.45	−0.002 (−0.007 to 0.004)	.52	−0.003 (−0.010 to 0.004)	.46
Adult SES	0.254 (0.235 to 0.274)	<.001	0.329 (0.303 to 0.355)	<.001	0.126 (0.099 to 0.152)	<.001
Adult SES × time	0.012 (0.008 to 0.016)	<.001	0.012 (0.006 to 0.017)	<.001	0.014 (0.007 to 0.021)	<.001
Lifetime SES	0.281 (0.261 to 0.302)	<.001	0.369 (0.342 to 0.400)	<.001	0.0134 (0.116 to 0.170)	<.001
Lifetime SES × time	0.012 (0.008 to 0.016)	<.001	0.012 (0.007 to 0.017)	<.001	0.012 (0.005 to 0.019)	<.001

Abbreviation: SES, socioeconomic status.

^a^ Please note that models with child SES and adult SES included both variables. The models with lifetime SES included just lifetime SES. All SES units are measured in standardized units. All of the time interactions are annual rates of decline.

^b^ Models controlled for age, sex, race.

^c^ Stratified models controlled for age and sex.

Regarding the domains of cognition, higher SES in adulthood was associated with better episodic memory for both groups of older adults at baseline but not with a change in episodic memory over time for either group. Figure 2B illustrates this difference in episodic memory performance between high (90th percentile) and low SES (10th percentile) individuals in adulthood for both groups. Higher SES in adulthood was associated with better baseline speed scores in Black and White individuals. Over time, higher adult SES was not associated with a change in the speed score of White individuals. Conversely, higher SES in adulthood was associated with slower speed scores in Black individuals. For each unit increase in SES, the speed score declined annually by −0.013 (95% CI, −0.017 to −0.010). Figure 2C shows these differences in speed scores between high (90th percentile) and low SES (10th percentile) individuals in adulthood for both groups. Adult SES was associated with better MMSE scores for both groups at baseline and over time, as shown in Figure 2D.

### SES in Childhood

Higher childhood SES was associated with better global cognitive functioning in Black individuals at baseline but not with changes in cognition over time. In White individuals, there was no association between childhood SES and global cognition at baseline or over time. eFigure 1A in Supplement 1 visually represents the global cognitive functioning between high (90th percentile) and low SES (10th percentile) in childhood for both groups. For subdomains of cognitive function, there was no association between SES and episodic memory or SES and MMSE scores in Black individuals or White individuals at baseline or over time (eFigures 1B and 1D in Supplement 1). In Black individuals, higher SES in childhood was associated with faster processing speed at baseline, but not in White individuals. Childhood SES was not associated with a change in processing speed over time in either group of older adults (eFigure 1C in Supplement 1).

### Lifetime SES

For all the participants, lifetime SES was associated with global cognitive function at baseline (estimate, 0.337; 95% CI, 0.317 to 0.357; *P* < .001) but not with changes over time (estimate, 0.003; 95% CI, −0.001 to 0.006; *P* = .10), as shown in Table 2. For the domains of cognitive functioning, lifetime SES was similarly associated with baseline levels of episodic memory for all participants but not with changes in episodic memory over time. Conversely, higher lifetime SES was associated with higher baseline speed scores for all groups and decreased speed scores over time for Black individuals but not White individuals (eFigure 2C in Supplement 1). Higher lifetime SES was associated with higher MMSE scores at baseline (estimate, 0.281; 95% CI, 0.261 to 0.302; *P* < .001) and a slower decline over time in both groups (estimate, 0.012; 95% CI, 0.008 to 0.016; *P* < .001). See eFigures 2A to 2D in Supplement 1 for visual representations.

### Associations Between SES and Brain Regions

In the subset of 933 participants who underwent MRI, as demonstrated in Table 3, we found no associations between childhood SES and any other brain metric. Higher adult SES and lifetime SES were positively associated with total brain volume (estimate, 3.18; 95% CI, 0.20 to 6.17; *P* = .04) and a lower WMH burden (estimate, −0.11; 95% CI, −0.21 to −0.01; *P* = .03), indicating positive brain health outcomes. We found no association between hippocampal volume and SES at any time point.

**Table 3.** Association of SES and Brain Region in a Subset of Participants^a^

Variable	Childhood SES		Adult SES		Lifetime SES	
	Estimate (95% CI)	*P* value	Estimate (95% CI)	*P* value	Estimate (95% CI)	*P* value
Total brain volume (n = 933)	1.33 (−1.52 to 4.19)	.36	3.04 (0.33 to 5.75)	.03	3.18 (0.20 to 6.17)	.04
Hippocampal volumes (n = 933)	0.03 (−0.04 to 0.09)	.39	0.01 (−0.05 to 0.07)	.68	0.02 (−0.04 to 0.87)	.52
White matter hyperintensities (n = 888)	0.03 (−0.07 to 0.12)	.59	−0.13 (−0.22 to −0.04)	.004	−0.11 (−0.21 to −0.01)	.03

Abbreviation: SES, socioeconomic status.

^a^ Linear regression models controlled for age, sex, and race.

## Discussion

In this longitudinal cohort study, a clear finding was that higher lifetime SES was associated with better global cognitive functioning at baseline for all participants, with adulthood SES being a higher contributor than childhood SES. This finding is consistent with several other studies showing that resources in adulthood provide a cognitive fitness advantage.^3,4,5^ For Black individuals only, higher childhood SES was associated with better global cognitive functioning and speed scores at baseline, similar to other studies^6,37,38^ that reported an association between childhood SES and cognitive outcomes.

Our findings regarding cognitive decline indicated that lifetime SES was not associated with a decline in our global cognition summary measure or episodic memory. However, lifetime SES was associated with a slower decline in MMSE score—a measure of general cognitive function—for all participants. Higher lifetime SES was associated with better speed scores at baseline for all participants but declined over time in Black individuals. The reason for the different findings between groups is not apparent, suggesting that factors other than SES may differentially affect Black individuals compared with White individuals. For example, Zahodne et al^39^ reported that psychosocial factors may contribute to racial and ethnic differences in cognitive decline in addition to SES factors. The authors reported that SES contributed to only 50% of the racial and ethnic differences in cognition, and 5% of the disparity between Black individuals and White individuals was attributed to differences in external perceived control,^39^ indicating how much control a person feels over life’s outcomes. External perceived control is characterized by the feeling that life is beyond a person’s control or that they are being pushed around^40^ and is more likely to be experienced by Black individuals^41^ because of historical marginalization. Similarly, Avila and colleagues^42^ found that educational level reduced the negative impact of WMH on language and memory tests for White individuals, but not for Black or Hispanic individuals, concluding that cognitive reserve does not manifest in the same manner across racial and ethnic groups. They propose that quality of education^42^ and the intersectionality with adult opportunities and discrimination in the labor market are likely explanations for the racial and ethnic discrepancies.

Using National Health and Nutrition Examination Survey III data from the Centers for Medicare and Medicaid Services (1988-2014), Beydoun et al^43^ reported that SES mediated the association between race and dementia. The authors described 3 potential pathways leading to racial and ethnic and SES disparities in all-cause dementia in older US adults: (1) SES is associated with lifestyle, which is associated with dementia, with an indirect effect (IE) β of −0.041 (SE, 0.014; *P* = .004); (2) SES is associated with lifestyle, which is associated with cognition, which is associated with dementia, with an IE β of −0.006 (SE, 0.001; *P* < .001); and (3) SES is associated with cognition, which is associated with dementia, with an IE β of −0.040 (SE, 0.008; *P* < .001).

The lifestyle variables most associated with health care discrepancies were diet and social support.^43^ Our study did not examine potential pathways; however, the pathways outlined by Beydoun et al^43^ provide information on possible mechanisms that may apply to our results. The finding in our study that SES was associated with lower cognitive functioning at baseline but not with cognitive decline is consistent with the third pathway above. SES is associated with the level at which someone performs cognitive performance tests, thereby lowering the threshold to receive a dementia diagnosis. In other studies of the same cohort, lifestyle has been associated with the risk of dementia,^44^ which is generally consistent with the first pathway above. More research is needed to understand the pathways that apply best to our cohort and to identify the key factors influencing cognitive function and decline.

The MRI results also contribute to our understanding of the possible mechanisms of action responsible for the association between SES and cognitive functioning. We found that childhood SES was not associated with any particular brain characteristic, whereas higher adult SES and lifetime SES were associated with greater total brain volume and lower WMH. These findings are consistent with other studies^17,19^ that reported the associations between brain characteristics and SES. More importantly, these results mirrored our associations between SES and cognitive functioning. Higher adult and lifetime SES were associated with better cognitive functioning and indicators of a structurally healthier brain. We failed to find an association between hippocampal volume and SES at any time; however, studying brain characteristics with SES is an evolving body of literature. Although well studied in the context of neurological disease, hippocampal volume has been less studied in older adults living in the community without neurological diagnoses. A previous study^18^ revealed that lower SES was associated with faster hippocampal atrophy. The results of a memory training intervention in older adults in Norway revealed increased hippocampal volume in older adults after 10 weeks of active training,^45^ suggesting a link between size and environmental exposure. These studies illustrate the need for more research on the dynamic nature of hippocampal volume and its association with cognitive function.

The present study has numerous strengths. We measured SES at 3 time points—in childhood, adulthood, and across the lifespan—allowing us to examine the influence on brain health in a comprehensive way. We examined a large number of older adults using cognitive functioning and brain structure outcomes. Black individuals, who had lower SES at all time points, were well represented in our study. Our study adds to the growing body of literature that examines SES in the context of brain health and provides data on MRI biomarkers and their association with SES in a group of diverse older adults.

### Limitations

The present study also has several limitations. Our measures of SES may not have been adequate to fully captured brain health inequities. We did not look at wealth or nonhousing wealth,^46^ which are important factors in examining brain health equity. Specifically, with increasing age, wealth explains more of the discrepancy between Black and White populations than income does,^13^ and we may have failed to capture the influences of all SES variables, in particular within the context of different racial and ethnic groups, such as quality of education. Furthermore, our measure of SES required participants to self-report current and historical information. Accurate recollection of SES may have been affected by several issues, including undiagnosed incidental cognitive impairment, despite having excluded persons with dementia diagnoses at baseline.

## Conclusions

In this cohort study, we found that SES, mainly in adulthood, was likely universally associated with brain health inequities, primarily through its influence on how one arrives at older adulthood. There was evidence that lifetime SES was associated with the rate of decline in specific cognitive tests (eg, MMSE). Adult SES and lifespan SES were associated with brain characteristics (WMH and total brain volume), suggesting a potential mechanism of action. Finally, because SES was higher for White individuals than for Black individuals at all time points, it is imperative to focus on Black individuals with lower SES to address brain health inequities. Additionally, it will be important to deepen our understanding of how SES differentially interacts with other psychosocial variables, such as perceived control and life course factors, such as quality of education.

## Notes

Supplement 1. **eFigure 1.** Longitudinal change in global cognitive function for Non-Hispanic African Americans and Non-Hispanic Whites stratified by adulthood SES (10^th^ and 90^th^ percentiles) **eFigure 2.** Longitudinal change in global cognitive function for Non-Hispanic African Americans and Non-Hispanic Whites stratified by lifetime SES (10^th^ and 90^th^ percentiles)

Supplement 2.
Data Sharing Statement
