# Supplementary material for: Lifetime Socioeconomic Status, Cognitive Decline, and Brain Characteristics
Source: JAMA Netw Open. 2025 Feb 21;8(2):e2461208. doi: 10.1001/jamanetworkopen.2024.61208 (PMC11846010; doi:10.1001/jamanetworkopen.2024.61208)
Supplement: Supplement 1. — eFigure 1. Longitudinal change in global cognitive function for Non-Hispanic African Americans and Non-Hispanic Whites stratified by adulthood SES (10th and 90th percentiles) eFigure 2. Longitudinal change in global cognitive function for Non-Hispanic African Americans and Non-Hispanic Whites stratified by lifetime SES (10th and 90th percentiles) [file jamanetwopen-e2461208-s001.pdf]

Supplemental Online Content

Krueger KR, Desai P, Beck T, et al. Lifetime socioeconomic status, cognitive decline, and brain characteristics. *JAMA Netw Open*. 2025;8(2):e2461208. doi:10.1001/jamanetworkopen.2024.61208

eFigure 1. Longitudinal change in global cognitive function for Non-Hispanic African Americans and Non-Hispanic Whites stratified by adulthood SES (10th and 90th percentiles)
eFigure 2. Longitudinal change in global cognitive function for Non-Hispanic African Americans and Non-Hispanic Whites stratified by lifetime SES (10th and 90th percentiles)

This supplemental material has been provided by the authors to give readers additional information about their work.

© 2025 Krueger KR et al. *JAMA Network Open*.

eFigure 1. Longitudinal change in global cognitive function for Non-Hispanic African Americans and Non-Hispanic Whites stratified by adulthood SES (10th and 90th percentiles)

Figure 1 Legend
Figure 1a: Global cognition – Adult SES
Figure 1b: Episodic Memory – Adult SES
Figure 1c: Speed Score – Adult SES
Figure 1d: MMSE Score – Adult SES

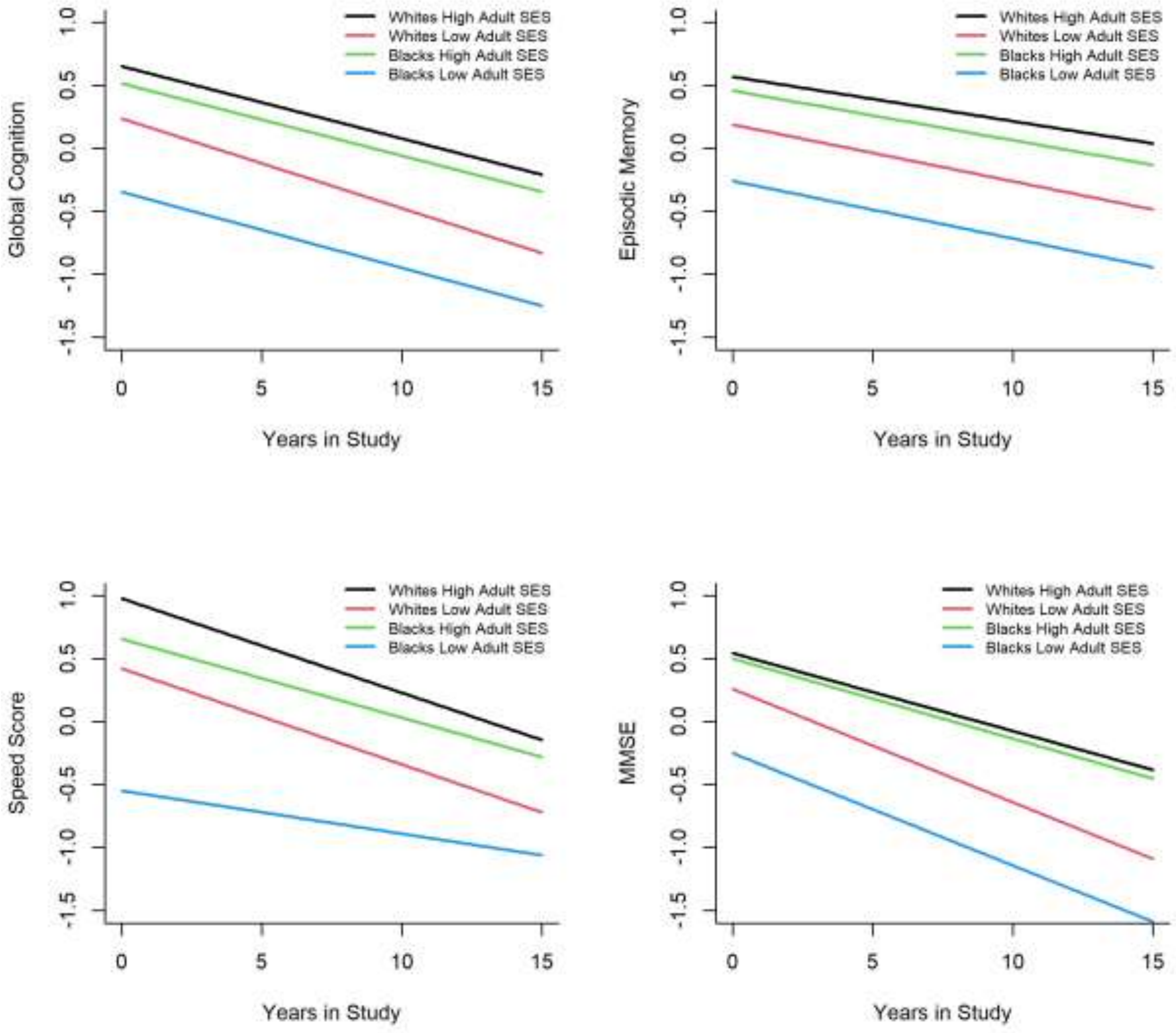

© 2025 Krueger KR et al. *JAMA Network Open*.

eFigure 2. Longitudinal change in global cognitive function for Non-Hispanic African Americans and Non-Hispanic Whites stratified by lifetime SES (10th and 90th percentiles)

<u>Figure 2 Legend</u>
Figure 2a: Global cognition – Lifetime SES
Figure 2b: Episodic Memory – Lifetime SES
Figure 2c: Speed Score – Lifetime SES
Figure 2d: MMSE Score – Lifetime SES

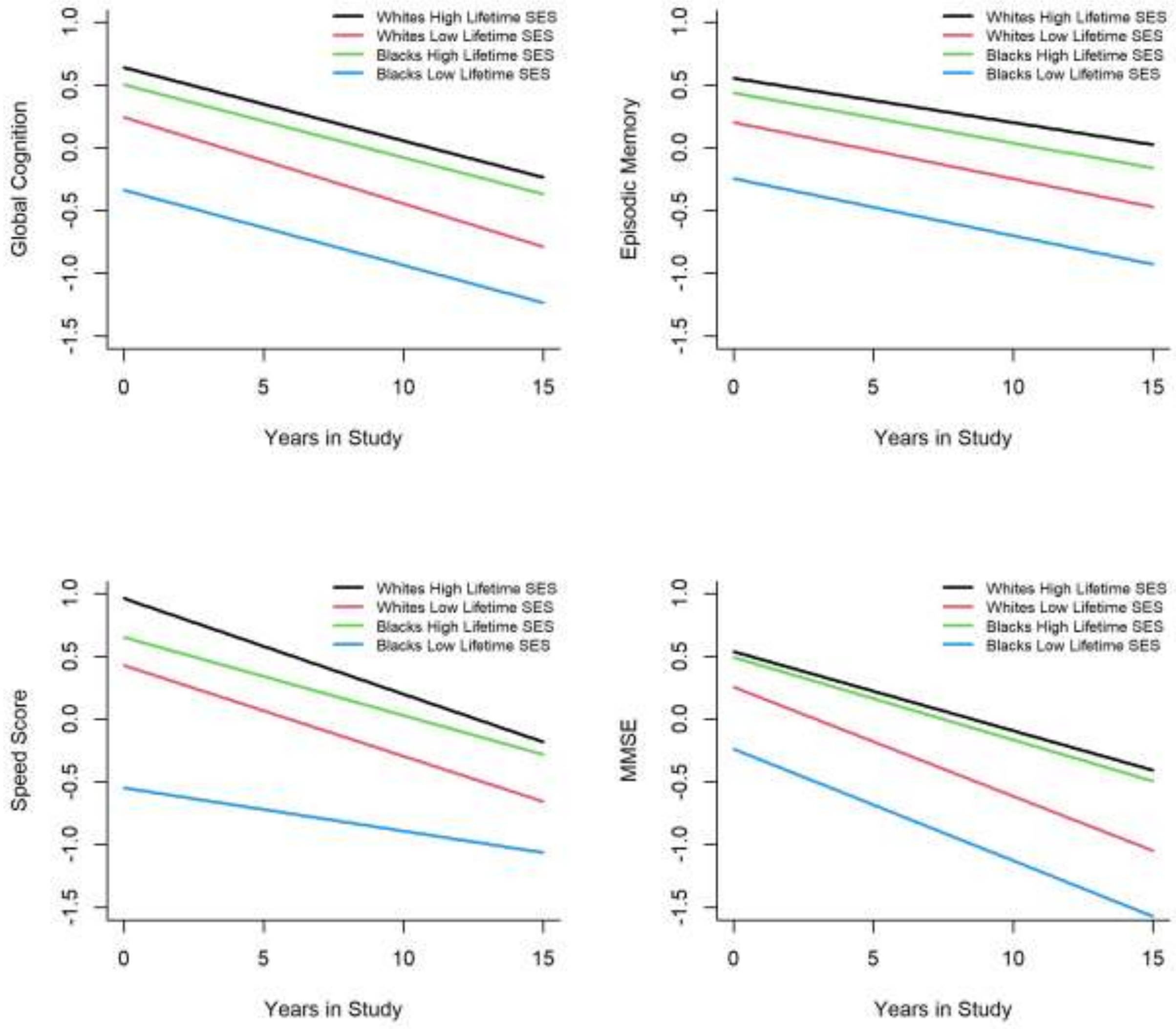

© 2025 Krueger KR et al. *JAMA Network Open*.
