# Supplementary material for: Lifetime Socioeconomic Status, Cognitive Decline, and Brain Characteristics
Source: JAMA Netw Open. 2025 Feb 21;8(2):e2461208. doi: 10.1001/jamanetworkopen.2024.61208 (PMC11846010; doi:10.1001/jamanetworkopen.2024.61208)
Supplement: Supplement 2. — Data Sharing Statement [file jamanetwopen-e2461208-s002.pdf]

Data Sharing Statement

Krueger. Lifetime Socioeconomic Status, Cognitive Decline, and Brain Characteristics. *JAMA Netw Open*. Published February 21, 2025. doi:10.1001/jamanetworkopen.2024.61208

Data
Data available: Yes
Data types: Deidentified participant data, Data dictionary
How to access data: Open Science Statement: Requests for data, analytic methods, and study materials can be submitted here: RIHA (rush.edu), and each request will be individually reviewed.
When available: With publication

Supporting Documents
Document types: None

Additional Information
Who can access the data: Researchers whose proposed use of the data has been approved.
Types of analyses: For specified purposes.
Mechanisms of data availability: After approval of a proposal.
